# Long-term clinical outcomes in patients with CIS treated with interferon beta-1b: results from the 15-year follow up of the BENEFIT trial

**DOI:** 10.1007/s00415-024-12417-x

**Published:** 2024-05-10

**Authors:** Ludwig Kappos, Gilles Edan, Mark S. Freedman, Hans-Peter Hartung, Xavier Montalbán, Frederik Barkhof, Ralf Koelbach, David G. MacManus, Eva-Maria Wicklein, for the BENEFIT and BENEFIT 15 Study Groups

**Affiliations:** 1 Research Center for Clinical Neuroimmunology and Neuroscience Basel (RC2NB), Departments of Head-Organs, Spine and Neuromedicine, Clinical Research, Biomedicine and Biomedical Engineering, University Hospital and University of Basel, Spitalstrasse 2, 4031 Basel, Switzerland; 2 CHU Hôpital Pontchaillou, Rennes, France; 3 Department of Medicine, University of Ottawa and The Ottawa Hospital Research Institute, Ottawa, Canada; 4 Department of Neurology, Medical Faculty, Heinrich-Heine Universität, Düsseldorf, Germany; 5 Multiple Sclerosis Center of Catalonia (Cemcat), Hospital Universitari Vall d’Hebron, Barcelona, Spain; 6 Amsterdam UMC, Location Vrije Universiteit, Amsterdam, The Netherlands; 7 Queen Square Institute of Neurology and Centre for Medical Image Computing, UCL, London, UK; 8 Ingress-Health HWM GmbH, Wismar, Germany; 9 UCL Queen Square Institute of Neurology, London, UK; 10 Bayer AG, Berlin, Germany; 11 Brain and Mind Center, University of Sydney, Sydney, Australia; 12 Department of Neurology, Palacky University in Olomouc, Olomouc, Czech Republic; 13 Department of Neurology, Medical University of Vienna, Vienna, Austria

**Keywords:** Multiple sclerosis, Interferon beta-1b, Clinically isolated syndrome, Immunotherapy

## Abstract

Multiple sclerosis (MS) treatment intervention with immunomodulating therapy at early disease stage improves short term clinical outcomes. The objective of this study is to describe the long-term outcomes and healthcare utilization of patients with clinically isolated syndrome (CIS) included in the Betaferon®/Betaseron® in Newly Emerging MS for Initial Treatment (BENEFIT) randomized, parallel group trial. In BENEFIT patients were assigned to “early” IFNB-1b treatment or placebo (“delayed” treatment). After 2 years or conversion to clinically definite multiple sclerosis (CDMS), all patients were offered IFNB-1b and were reassessed 15 years later. Of 468 patients, 261 (55.8%) were enrolled into BENEFIT 15 (161 [55.1%] from the early, 100 [56.8%] from the delayed treatment arm). In the full BENEFIT analysis set, risk of conversion to CDMS remained lower in the early treatment group ( – 30.5%; hazard ratio 0.695 [95% CI, 0.547–0.883]; *p* = 0.0029) with a 15.7% lower risk of relapse than in the delayed treatment group (*p* = 0.1008). Overall, 25 patients (9.6%; 9.9% early, 9.0% delayed) converted to secondary progressive multiple sclerosis. Disability remained low and stable with no significant difference between groups in Expanded Disability Status Scale score or MRI metrics. Paced Auditory Serial Addition Task-3 scores were better in the early treatment group (*p* = 0.0036 for treatment effect over 15 years). 66.3% of patients were still employed at Year 15 versus 74.7% at baseline. In conclusion, results 15 years from initial randomization support long-term benefits of early treatment with IFNB-1b.

## Introduction

Multiple sclerosis (MS) is the most common cause of chronic neurological disability in young adults [1, 2]. Over the past 25 years, increased understanding of MS and availability of disease-modifying therapies (DMTs) has improved options to manage the disease [1]. Typically MS lasts several decades [2–4]. Prolonged follow-up of well characterized patients provides critical information on the implications of early treatment intervention.

The Betaferon®/Betaseron® in Newly Emerging MS for Initial Treatment (BENEFIT) trial investigated the effects of interferon beta-1b (IFNB-1b) treatment at or shortly after clinically isolated syndrome (CIS)—the first neurological episode suggestive of MS—on clinical and MRI outcomes [5–8]. The BENEFIT trial has been recognized as the most comprehensive follow-up study of patients who received early intervention with IFNB-1b, and has contributed to the broad consensus to offer patients early treatment to optimize disease management [1, 9]. Early study results demonstrated that initiating interferon beta-1b at CIS, compared with those who started treatment after a first relapse or after two years was associated with improved clinical and MRI outcomes beyond the 2-year double-blind randomized trial and through the 5-year rater-blinded study period.

Additional follow-up with a prospective, cross-sectional assessment at 11 years post baseline showed that early treatment with IFNB-1b remained beneficial up to 11 years after randomization. Patients who started IFNB-1b at CIS retained a lower risk for conversion to clinically definite multiple sclerosis (CDMS) and a lower annualized relapse rate (ARR) compared with those who started IFNB-1b after a short delay (mean 1.5 years) [5]. Disability remained low and stable in both treatment groups at the 11-year assessment.

This study aims to further extend the long-term information on this cohort and describe the course of this well-characterized inception cohort by an additional prospectively planned assessment 15 years after randomization. Data is reported from patient visits at Year 15 and from integrated analyses of the full BENEFIT cohort.

## Methods

Patients with CIS and ≥ 2 brain MRI lesions suggestive of MS were randomly assigned in a 5:3 ratio to receive IFNB-1b (“early treatment”) or placebo. [8] After conversion to CDMS (defined as a second clinical attack or confirmed disease worsening), patients were offered open-label treatment with IFNB-1b without disclosing the initial randomization. At 2 years, all patients (including those who had not converted to CDMS) were offered to continue taking interferon beta-1b. The “delayed treatment” group included patients who were initially randomized to placebo. IFNB-1b was administered at a dose of 250 µg subcutaneously, every other day.

Prospective assessments (blinded as for the initial randomization) continued for 5 years following randomization. At 15 years, all initially randomized patients were asked to participate in a comprehensive clinical and MRI reassessment. Patients could be evaluated at their original center or, if their original study center did not participate in BENEFIT 15, at a different, participating center. Clinical outcomes included assessment of conversion to CDMS, progression to secondary progressive multiple sclerosis (SPMS), ARR, Expanded Disability Status Scale (EDSS), Paced Auditory Serial Addition Task – 3 s (PASAT-3), and employment status. CDMS was reached if a new neurological event (relapse) occurred, ie, the appearance of new neurological abnormality or reappearance of a neurological abnormality, separated by at least 30 days from onset of a preceding clinical demyelinating event, or by sustained worsening of >  = 1.5 points on the EDSS and a total EDSS of >  = 2.5. HRQoL was assessed by EuroQol 5-Dimension (EQ-5D), including the Visual Analog Scale (VAS) and Functional Assessment of Multiple Sclerosis (FAMS – total score and Trial Outcome Index (TOI)). Standardized questions on employment situation and resource utilization were asked. If a visit at the study center was not possible, patients were offered a structured telephone evaluation that also included a validated patient reported EDSS assessment tool for which an overall high correlation with EDSS as determined by physical examination had been demonstrated (Pearson’s correlation coefficient -0.95), across all functional scores and degrees of disability.[10, 11].

Investigators collected MRI data at study sites according to the BENEFIT MRI standardized protocol. Scans were analyzed at a central reading site (Institutes of Neurology and Healthcare Engineering, UCL, London, UK). Trained readers manually identified and quantified lesions using a local-intensity thresholding technique. Neuroradiological assessments included number of new T2 lesions, T2 lesion volume, T1 lesion volume, normalized brain volume, normalized thalamic volume, cortical thickness, and mean upper cervical cord area [5]*.*

Analyses were performed for the BENEFIT 15 cohort. Integrated analyses were carried out using the full BENEFIT cohort, allowing for time to event calculations. Outcomes included time to conversion to CDMS, time to first relapse, time to recurrent relapse, number of patients with confirmed and sustained 1-point EDSS progression, and number of patients with confirmed 2.5-point EDSS progression. The number of patients with diagnosis of MS according to the McDonald 2001 and 2010 criteria at fifteen years after occurrence of CIS was determined in patients who had an MRI performed at Year 15. Patients who developed CDMS or McDonald MS by Year 11 of follow-up were also included in this analysis.

Safety was also assessed at Year 15. An adverse event (AE) was defined as any untoward medical occurrence (ie, any unfavorable and unintended sign, symptom, or disease temporally associated with the use of a medicinal product, whether or not considered related to the medicinal product, also covering laboratory findings or results of other diagnostic procedures considered clinically relevant). Conditions were documented that started or deteriorated after signing of informed consent.

### Statistical procedures

Statistical modeling was used to estimate treatment effects, and to explore the relationship between target variables and treatment. The study was exploratory in nature, with conversion to CDMS and/or SPMS, relapse rate, EDSS, PASAT-3, resource utilization, and employment/retirement status defined as variables of primary interest. All variables were analyzed descriptively with appropriate statistical methods. Particular outcomes (time to CDMS, time to first relapse, time to use of an ambulatory device, and time to dependence on an ambulatory device) were evaluated using Kaplan–Meier (KM) methods, log-rank tests and proportional hazards regression for time-to-event outcomes and a generalized linear regression model for ARR, with steroid use during the first event (yes or no); multifocal or monofocal onset of disease, number of T2 lesions at screening (2 to 4, 5 to 8, or ≥ 9); number of gadolinium-enhancing (Gd +) lesions at screening; age; and sex included as the set of covariates in the proportional hazards regression model and the generalized linear regression model. For PASAT-3 distribution over time a parametric longitudinal linear mixed model (with baseline PASAT-3 as the covariate) was established. The statistical evaluation was performed using software package SAS® (Statistical Analysis System) release 9.2 (SAS Institute Inc., Cary, North Carolina, United States).

### Standard protocol approvals, registrations, and patient consents

The institutional review boards of participating institutions approved the protocol for the study. Patients provided informed consent at enrollment of the trial. The BENEFIT 15 trial is listed on clinicaltrials.gov under NCT03269175.

## Results

### Patient disposition

**Fig. 1:**
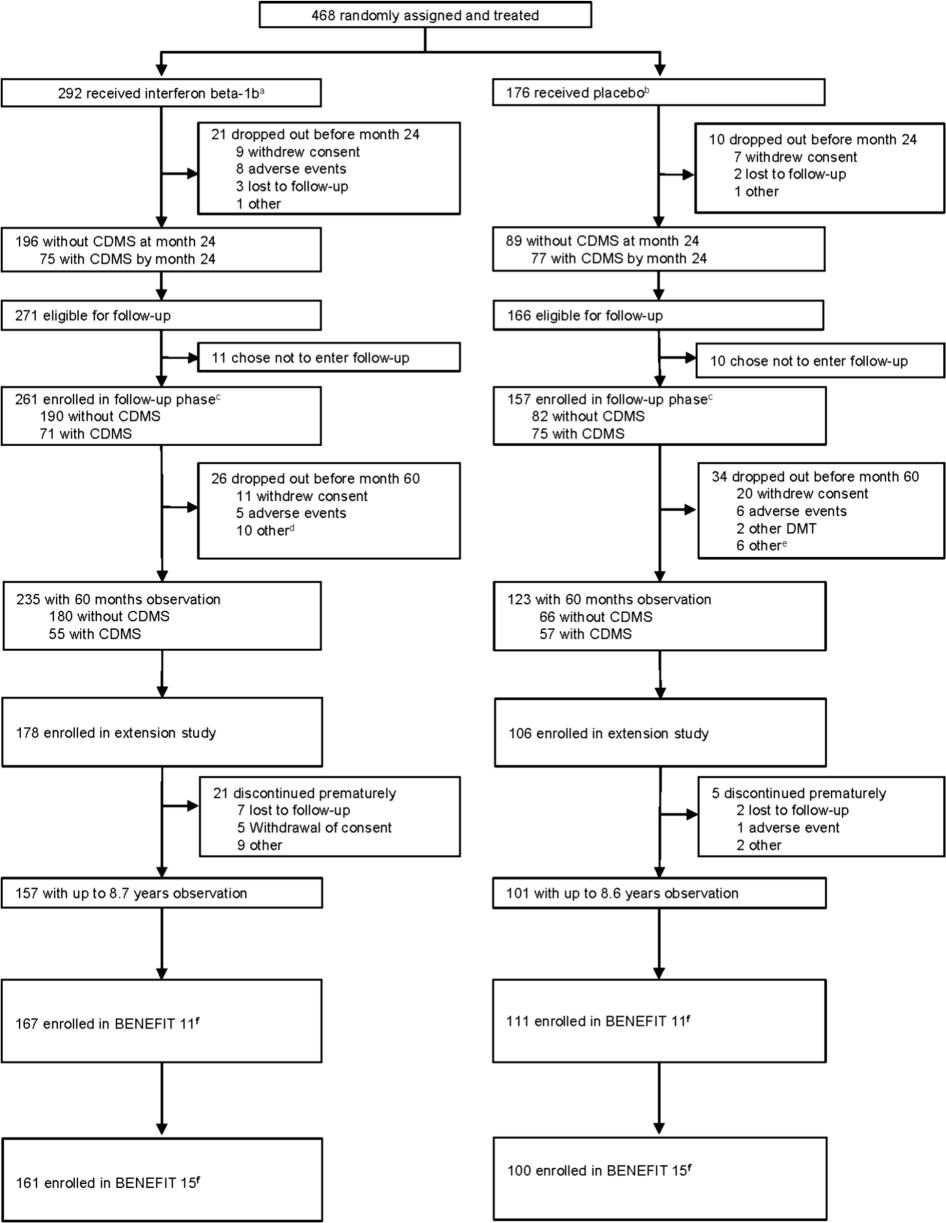
Study profile for the entire BENEFIT Study. **a** Includes one patient randomized to receive interferon beta-1b but treated with placebo. **b** Includes one patient randomized to receive placebo but treated with interferon beta-1b. **c** Includes one patient entered into the BENEFIT follow-up study after premature discontinuation of the BENEFIT Study. **d** Four lost to follow-up, 2 missing data, 1 noncompliance, 1 treatment failure, 2 refused final visit. **e** Three lost to follow-up, 1 relocated away from site, 1 pregnancy, 1 unable to attend visit because of job. **f** To be eligible for the 11-year or the 15-year cross-sectional follow-up study, patients only needed to be randomized and treated in the original BENEFIT Study (i.e., they did not need to be included in the previous BENEFIT analyses). *BENEFIT*  Betaferon/Betaseron in Newly Emerging MS for Initial Treatment, *CDMS*  clinically definite multiple sclerosis, *DMT*  disease-modifying therapy

Of the 468 patients initially randomized in the BENEFIT trial, 261 (55.8%) were enrolled in BENEFIT 15 (early treatment: 161 of 292 patients [55.1%], delayed treatment: 100 of 176 patients [56.8%]) between September 2017 and May 2018 at the 69 sites contributing in this 15-year follow-up study (Fig. 1). Sites included those actively participating in the BENEFIT 15 trial, as well as non-participating sites who had their patients enrolled for assessments at one of the active sites. Overall, 66.4% of the patients originally randomized and treated in these participating sites were included. Of the 261 patients enrolled in BENEFIT 15, a total of 199 patients (76.25%) attended the study centers in person; 62 patients (23.75%) were unable to attend study centers and were instead evaluated remotely by telephone interview.

The baseline characteristics of participants in the 15-year follow-up study did not differ from those in the initial BENEFIT trial cohort (Table 1). At the 15-year visit, 161 patients (61.7%; 61.5% of the early, 62% of the delayed group) were receiving a DMT, of which 37.3% (n = 60) were administering IFNB, with no difference in current use observed between the randomization groups (early treatment: 37,4%, delayed: 37.10%). Also escalation therapy use at the time of the study visit was similar (Step 1 escalation in 16.8% (n = 27) of the early and in 16.1% (n = 16) of the delayed treatment group, Step 2 escalation in 6.2% (n = 10) of the early and 8% (n = 8) of the delayed treatment group) Any previous or current Step 1 escalation therapy use was recorded for 29.2% (n = 47) of the patients in the early and by 25% (n = 25) in the delayed treatment group, and any Step 2 escalation therapy use for 14.9% of the early and 16% of the delayed treatment group. Mean follow-up time was 15.2 years and the mean (standard deviation [SD]) age in this cohort was 46.7 (7.3) years, with 8 patients (3.1%) ≥ 60 years of age. The mean delay in initiation of therapy in the delayed treatment group was 1.53 years.

**Table 1:** Baseline characteristics of the original BENEFIT population and the BENEFIT 15 population

	Original BENEFIT population			BENEFIT 15 population		
	Early treatment	Delayed treatment	Overall	Early treatment	Delayed treatment	Overall
n (% of original BENEFIT population)	292 (100)	176 (100)	468 (100)	161 (55.1)	100 (56.8)	261 (55.8)
Age (years), median (Q1-Q3)	30.0 (24.0–37.0)	30.0 (25.0–36.0)	30.0 (24.0–37.0)	30.0 (24.0–37.0)	30.0 (25.0–36.0)	30.0 (25.0–36.0)
Female, n (%)	208 (71.2)	123 (69.9)	331 (70.7)	115 (71.4)	69 (69.0)	184 (70.5)
EDSS at baseline, median (mean), Q1-Q3	1.50 (1.59), 1.00–2.00	1.50 (1.49), 1.00–2.00	1.50 (1.55), 1.00–2.00	1.50 (1.50), 1.00–2.00	1.50 (1.55), 1.00–2.00	1.50 (1.52), 1.00–2.00
Steroid use at CIS, n (%)	210 (71.9)	122 (69.3)	332 (70.9)	114 (70.8)	71 (71.0)	185 (70.9)
Number of T2 lesions, median (Q1, Q3)	18.0 (7.0–38.5)	17.0 (7.0–36.5)	17 (7.0–38.0)	19.0 (8.0–40.0)	17 (8.0–35.0)	18.0 (8.0–39.0)

*CIS* clinically isolated syndrome, *EDSS* Expanded Disability Status Scale, *Q1* first quarter, *Q3* third quarter

### Clinical outcomes

**Fig. 2:**
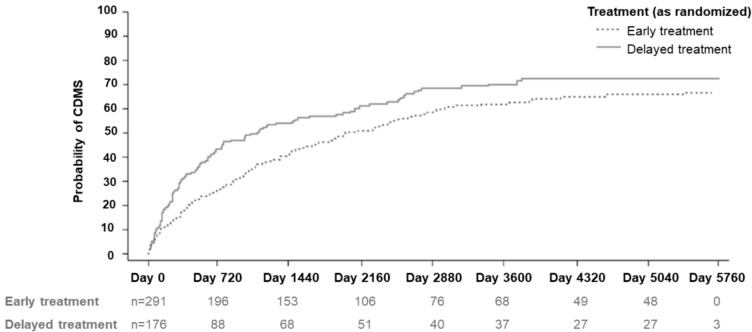
Kaplan–Meier estimates for probability of CDMS. *CDMS* clinically definite multiple sclerosis

Over the 15-year study period, the risk of conversion to CDMS among patients in the early treatment arm was 30.5% lower compared with the delayed treatment arm (hazard ratio 0.695 [95% confidence interval, 0.547–0.883] *p* = 0.0029). Kaplan–Meier (KM) estimate for conversion to CDMS by Year 15 was 69.8% in the overall study cohort (N = 468). Per KM estimate, 169 patients in the early treatment group (67.6% of the total early treatment group) and 118 patients in the delayed treatment group (73.5% of the total delayed treatment group) had converted to CDMS until Year 15 (Fig. 2). The risk of relapse was 15.7% lower in the early relative to the delayed treatment group (*p* = 0.1008). The mean ARR in the full BENEFIT cohort analysis set was 0.2083 over 15 years, and the median time to first relapse was 1888 days in the early treatment group compared to 931 days in the delayed treatment group. There was no significant difference between treatment groups regarding time to recurrent relapse (hazard ratio 0.842 [95% confidence interval, 0.671–1.056]).

In the BENEFIT 15 cohort, the KM estimate for the rate of conversion to SPMS at 15 years was 10.0% overall. No differences were observed in rates of conversion to SPMS between the two treatment groups (early treatment: 16/161 patients [9.9%, KM estimate: 10.2%]; delayed treatment: 9/100 patients [9.0%, KM estimate: 9.9%]; log-rank test *p* = 0.9713). SPMS occurrence was weakly associated with higher age (HR 1.056 [p = 0.0484]. In the delayed treatment group, KM estimate for SPMS was 4.0% for younger patients (< 30 years at study entry) versus 14.3% for those who entered BENEFIT at the age of 30 years or above. However, in the early treatment group, KM estimate for conversion to SPMS in patients below 30 years of age at BENEFIT study entry was similar (10.5%) to patients >  = 30 years (9.9%).

**Fig. 3:**
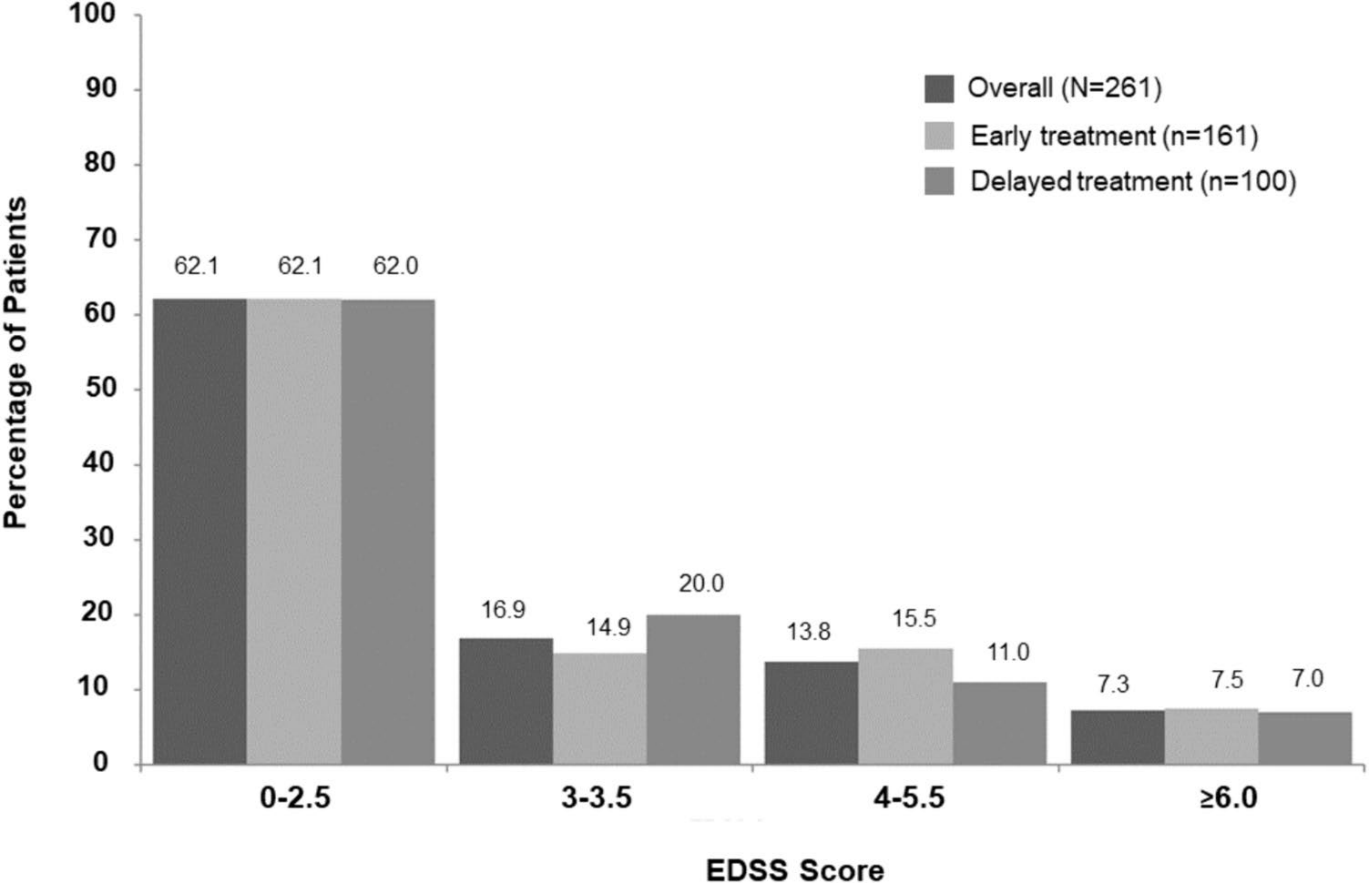
EDSS scores in the BENEFIT 15 population. *EDSS* expanded disability status scale

At the Year 15 follow-up assessment, the mean (SD) EDSS score was 2.5 (1.76), and the median EDSS score (Q1, Q3) was 2.0 (1.5, 3.5). The mean (SD) EDSS scores in the early treatment and delayed treatment groups were 2.55 (1.76) and 2.43 (1.76), respectively; the median EDSS score [Q1, Q3] was 2.0 (1.5, 3.5) and 2.0 (1.0, 3.5), respectively. Overall, at the Year 15 follow-up, the majority of patients in both treatment arms presented with minimal disability, corresponding to an EDSS score of ≤ 2.5 (Fig. 3); 2.7% of the patients participating in the 15-year follow-up had an EDSS of ≥ 7 and depended on using a wheelchair as mobility aid. The numbers of patients with confirmed and sustained 1-point EDSS worsening were 91 (56.5%) and 47 (29.2%), respectively, in the early treatment group and 49 (49%) and 34 (34%), respectively, in the delayed treatment group. Within the same cohort, 32 (19.9%) patients had confirmed 2.5-point EDSS worsening in the early treatment group compared to 18 (18%) patients in the delayed treatment group.

**Fig. 4:**
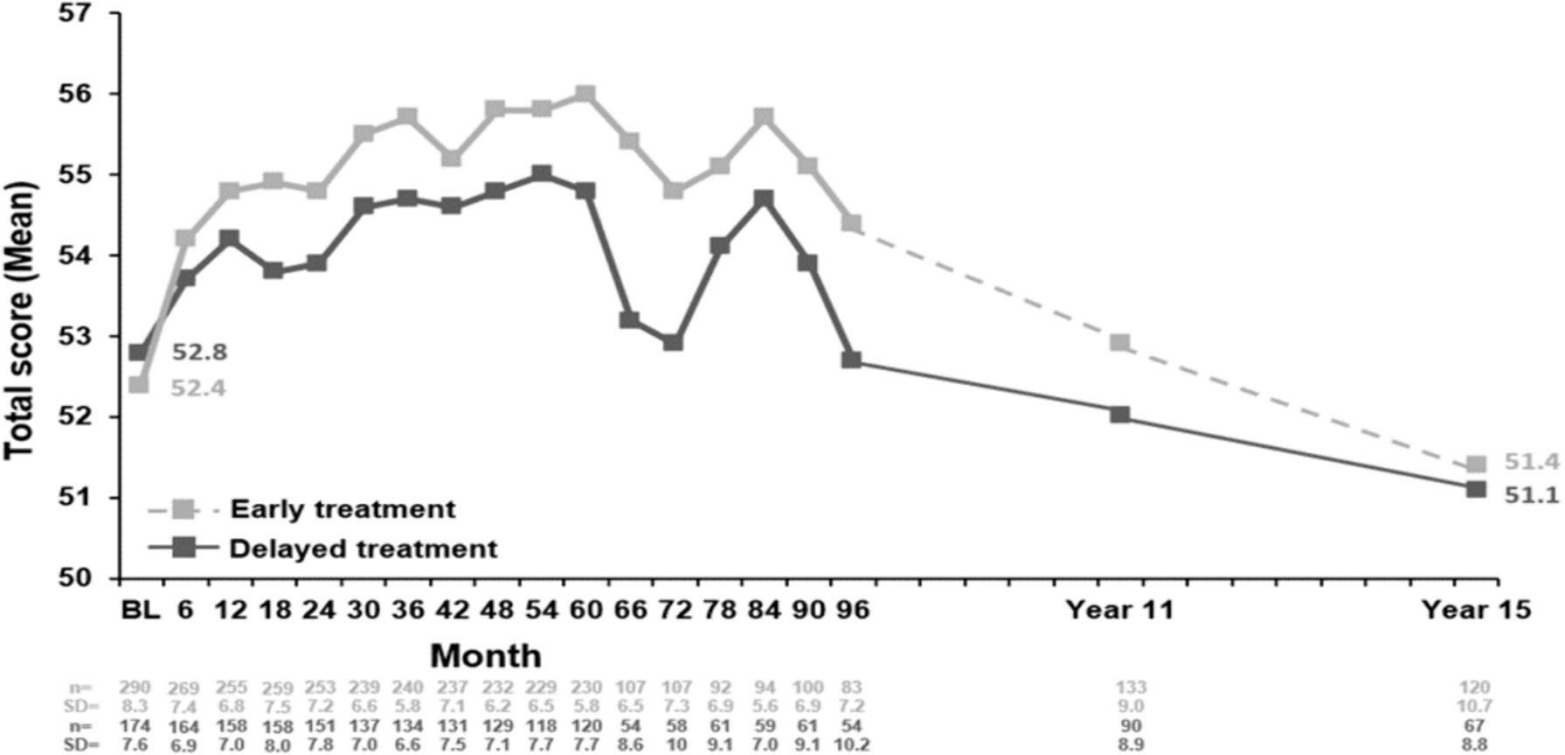
PASAT-3 score over 15 years in both early and delayed treatment groups. BL, baseline; PASAT-3, Paced Auditory Serial Addition Task – 3 s; SD, standard deviation

Mean (SD) PASAT-3 score at Year 15 was similar in both treatment groups: 51.4 (10.7) in the early treatment group and 51.1 (8.8) in the delayed treatment group. The median (Q1, Q3) PASAT-3 score at 15 years after the subject´s first clinical event was 55.0 (50.0–58.0) in the early treatment group and 54.0 (49.0–57.0) in the delayed treatment group. For PASAT-3 score over the 15-year period a positive treatment effect could be observed (*p* = 0.0036 for treatment effect, adjusted for baseline PASAT score; Fig. 4).

### HRQoL outcomes

**Fig. 5:**
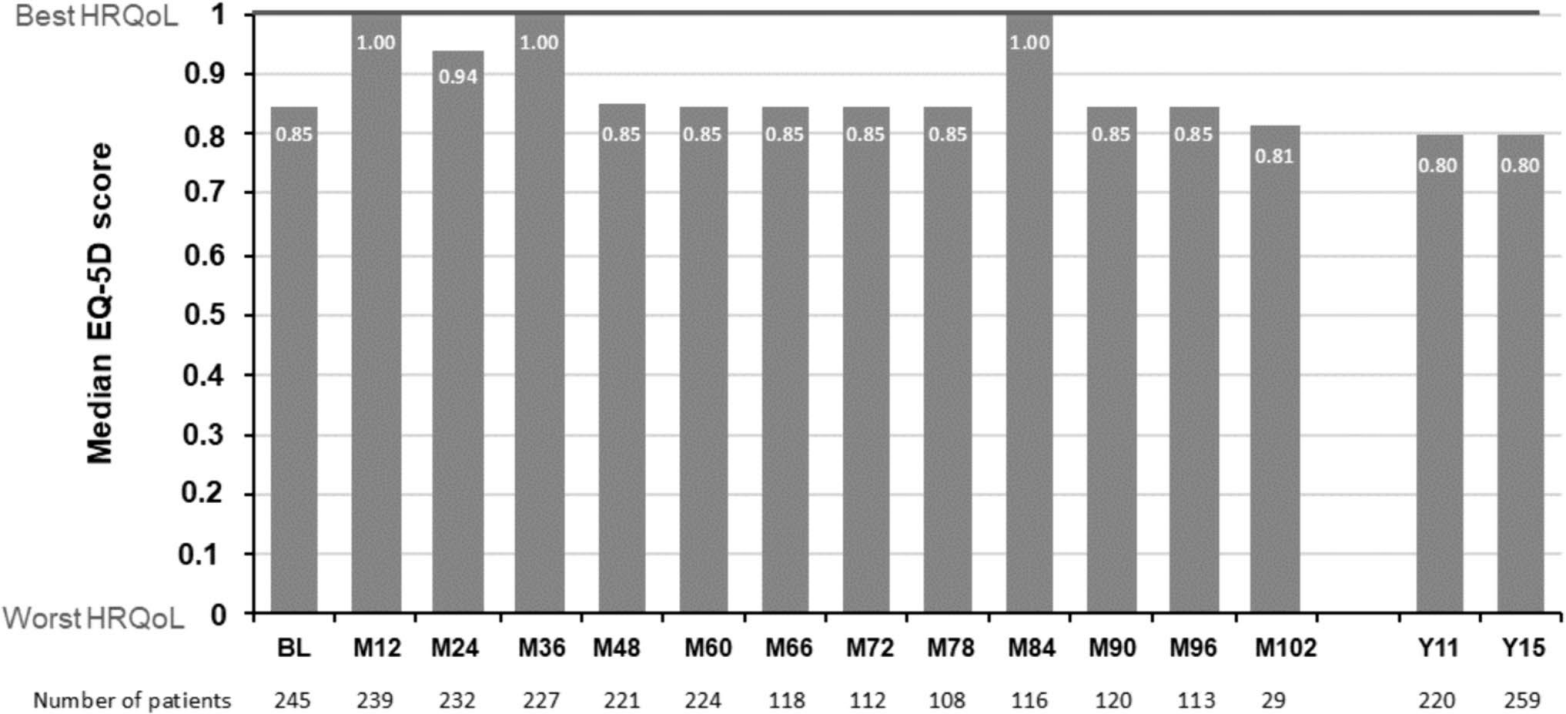
Median EQ-5D score in the overall BENEFIT 15 cohort. *BL* baseline, *EQ-5D* EuroQol 5-Dimension, *HRQoL* health-related quality of life, *M* month, *Y* year

At Year 15, the median (Q1, Q3) EQ-5D index score was 0.7960 (0.6910, 1.000; n = 259) in the overall cohort (Fig. 5). Median (Q1, Q3) EQ-5D change from baseline was 0.00 ( – 0.1880, 0.00). The EQ-5D VAS scores showed a similar pattern (85.50 [75.00, 93.00] at baseline (n = 186) and at Year 15 (n = 199; 80.00 [65.00, 90.00]). Although no statistical testing was conducted, the EQ-5D scores were generally similar between treatment groups. Median (Q1, Q3) FAMS-TOI was 114.50 (90.50, 133.00) at Year 15 (n = 260); median (Q1, Q3) change from baseline was  – 7 ( – 28, 2.54). FAMS total score (Q1, Q3) was 139.00 (111.50, 159.00) at Year 15 and the median (Q1, Q3) change from baseline was  – 5 ( – 27, 5.00).

### Employment and resource utilization

**Fig. 6:**
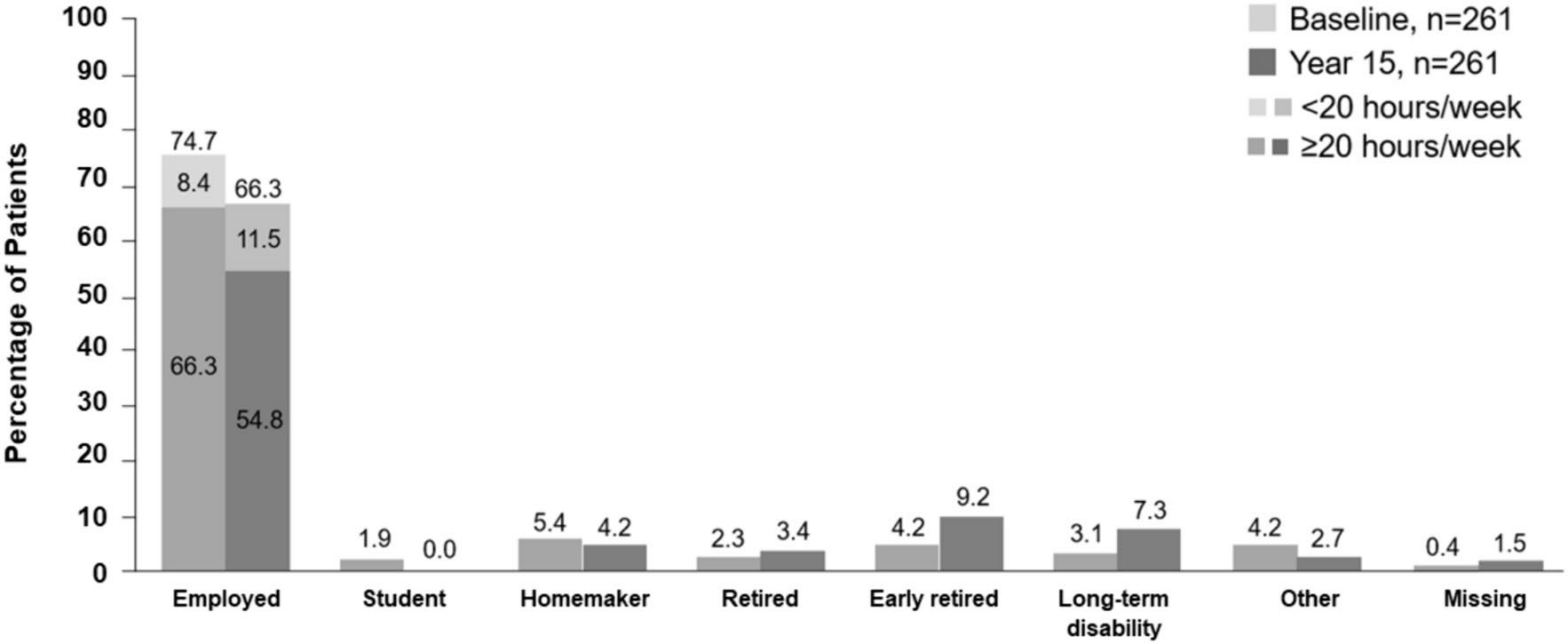
BENEFIT 15 patient cohort employment at baseline and Year 15

Employment status information was available for 257/261 patients (98.5%) participating in the BENEFIT 15 study. Overall, 173/261 patients (66.3%) remained employed at Year 15, compared to 195/261 (74.7%) employed at baseline (Fig. 6). At Year 15, 143 patients (54.8%) were working > 20 h per week, 30 patients (11.5%) were working < 20 h per week. A total of 155 patients (59.4%) reported MS as having no impact on their working ability and employment status and 162 patients (62.1%) reported experiencing no periods of being unable to work because of MS in the past 12 months. There were no reports of hospitalizations during the past 12 months in 245/261 patients (93.9%). The use of adaptations in the past 6 months because of MS were reported by 29 patients (11.1%) with walking aids being most commonly utilized (17 patients; 6.5%). Other adaptation included: wheelchairs (8 patients; 3.1%), special hygiene utensils (8 patients; 3.1%), adaptation for car (5 patients; 1.9%), ramps (4 patients, 1.5%), adaptation at work (4 patients, 1.5%), special kitchen utensils (4 patients, 1.5%), spectacles (2 patients, 0.8%), alarms (1 patient, 0.8%) and stair lift (1 patient, 0.4%). Employment and resource utilization were generally similar between the treatment groups.

### MRI Outcomes

MRI data were available for 110 patients (68.3%) in the early treatment group and 58 patients (58.0%) in the delayed treatment group. No consistent differences were seen between the treatment groups in MRI outcomes. The number of patients with any new T2 lesion since the patient’s last scan at Year 11 was 52 (47.3%) in the early and 30 (51.7%) in the delayed treatment arm. In the overall cohort, 41 patients (24.4%) experienced no new T2 lesions since their last analysis (data missing for 45 patients [26.8%]).

The McDonald criteria (2001 and 2010, respectively) were applied to identify patients who developed MS within 15 years after CIS. The 2010 McDonald criteria was applied to 255 patients (157 in the early treatment group, 98 in the delayed treatment group); in the early treatment group 149 (94.9%) met the criteria compared to 94 (95.9%) in the delayed treatment group. The 2001 McDonald criteria were applied in 252 patients (155 in the early treatment group, 97 in the delayed treatment group); 146 (94.2%) patients in the early treatment group met criteria compared to 92 (94.8%) in the delayed treatment group.

### Safety

None of the participants who received IFNB-1b therapy experienced an AE during the BENEFIT 15 study period, and none of the AEs reported in a previous BENEFIT study were ongoing at the BENEFIT 15 visit. No deaths, serious AEs, or pregnancies were reported during this study. Changes in vital signs and body mass index since BENEFIT study baseline were negligible.

## Discussion

BENEFIT 15 is a unique study cohort with 15-year follow-up data from randomization that compared early vs delayed treatment with IFNB-1b in patients presenting with CIS. Considering the time elapsed since the initial randomization, the number of patients enrolled in BENEFIT 15 is a respectable proportion—55.8%—of randomized patients, thus providing a unique opportunity to determine long-term outcomes of early treatment with IFNB-1b. The possibility that patients with a more severe disease course dropped out before this 15 year follow-up study cannot entirely be ruled out although such selective drop out may also have occurred with asymptomatic participants. However, the 15-year follow-up population did not appear to have a selection bias, as baseline characteristics were very similar to those of the original BENEFIT population, with a T2 lesion load at baseline that reflected substantial disease burden. Patients from sites that participated in this 15-year assessment were invited irrespective of their current medication, and great effort was made to reach more severely disabled patients with reduced mobility. In those cases where a clinic visit was not feasible, remote standardized telephone assessments were conducted. It is therefore remarkable that the conversion rates to CDMS after 15 years from initial randomization still favour the early treatment group (67.6% vs 73.5% with delayed treatment, by KM estimates). The overall relatively low EDSS and high PASAT-3 scores suggest limited disease progression by Year 15. EDSS scores compared favorably with natural history populations with shorter duration of follow-up [12, 13]. This, along with the low SPMS conversion rate after 15 years of follow up in both the early treatment and delayed but still early treatment groups, supports the overall beneficial effect of early initiation of DMTs. With an average of 1.5 years until the delayed treatment groups received treatment, both groups initiated treatment relatively early.

Patients in this study generally reported good HRQoL across both measures. Population surveys have demonstrated a relationship between age and HRQoL measures, and the mean EQ-5D VAS in the BENEFIT 15 cohort was comparable to that of EQ-5D VAS norm in an international general population for its respective age group [14].

Employment findings of the patients enrolled in the BENEFIT study at Year 15 reflect patients’ ability to accomplish daily professional tasks. These tasks often can be limited by MS symptoms and the disabilities inherent to the disease. The Global MS Employment Report demonstrated that 43% of unemployed patients with MS discontinued work within 3 years of diagnosis, and 70% after 10 years of diagnosis [15]. In fact, results from a prospective observational study showed that, in patients with MS, accumulation of disability and increase in relapse at 5 years following first episode of central nervous system demyelination were each associated with a decline in employment trajectory [16]. In the context of BENEFIT 15, the majority of patients (66.3%) were employed at Year 15 and had not taken days off from work in the past 12 months because of MS, regardless of whether they were in the early treatment or the delayed treatment arm of the initial BENEFIT cohort. This result supports the value of IFNB-1b treatment initiation at an early stage, especially when considering that the burden of MS can be substantial, throughout adult life.[17] In 2017, a survey of 4590 working-age patients with MS in Germany (mean (SD) age: 51.8 (11.0) years; mean (SD) age at diagnosis: 36.3 (10.6) years) revealed that 51% were employed or self-employed [18]. Despite their patient population having a shorter average disease duration, the findings from our study compare favorably with these results, with 66.3% of patients in our study being employed at Year 15.

Another survey of 1727 patients with MS in New Zealand, with a similar age and disease duration as our patient population, found that more than half (54%) were not working [19], while the study population in BENEFIT 15 including patients who either attended study centers or were evaluated via telephone interview had a non-employment rate of only 32.2%. This although great effort was taken to include all types of patients with varying disability, as patients with higher disability may be prone to non-employment. HRQoL data from the BENEFIT 15 study also compared favorably with population-based values and with data from other MS cohorts [20–22].

No new safety signals were detected with IFNB-1b long-term treatment in the current cross-sectional study. Clinical outcomes from the 15-year BENEFIT trial follow-up further support initiating DMT at or shortly after CIS. In the follow up of a placebo controlled CIS study comparing early interferon beta intramuscular treatment 10 years after randomization with delayed treatment Kinkel et al. reported that 18.4% had an EDSS ≥ 3.0.[23] In a study by Chung et al. conducted in a British cohort of untreated patients 30 years after CIS only 42% had remained ambulatory (EDSS scores of ≤ 3.5) and 34% developed SPMS [22].

With a follow-up of 15 years, the BENEFIT-15 cohort offers the longest duration of follow-up in a randomized population treated at or shortly after CIS, thus suggesting that many patients who start treatment with IFNB-1b at an early disease stage can retain clinical and social wellbeing in the future.

## Data Availability

Availability of the data underlying this publication will be determined according to Bayer’s commitment to the EFPIA/PhRMA “Principles for responsible clinical trial data sharing”. This pertains to scope, timepoint and process of data access. As such, Bayer commits to sharing upon request from qualified scientific and medical researchers’ patient-level clinical trial data, study-level clinical trial data, and protocols from clinical trials in patients for medicines and indications approved in the United States (US) and European Union (EU) as necessary for conducting legitimate research. This applies to data on new medicines and indications that have been approved by the EU and US regulatory agencies on or after January 01, 2014. Interested researchers can use www.vivli.org to request access to anonymized patient-level data and supporting documents from clinical studies to conduct further research that can help advance medical science or improve patient care. Information on the Bayer criteria for listing studies and other relevant information is provided in the member section of the portal. Data access will be granted to anonymized patient-level data, protocols and clinical study reports after approval by an independent scientific review panel. Bayer is not involved in the decisions made by the independent review panel. Bayer will take all necessary measures to ensure that patient privacy is safeguarded.
